# Methanethiol and Dimethylsulfide Cycling in Stiffkey Saltmarsh

**DOI:** 10.3389/fmicb.2019.01040

**Published:** 2019-05-10

**Authors:** Ornella Carrión, Jennifer Pratscher, Kumari Richa, Wayne G. Rostant, Muhammad Farhan Ul Haque, J. Colin Murrell, Jonathan D. Todd

**Affiliations:** ^1^School of Environmental Sciences, University of East Anglia, Norwich, United Kingdom; ^2^The Lyell Centre, Heriot-Watt University, Edinburgh, United Kingdom; ^3^School of the Environment, Florida Agricultural and Mechanical University, Tallahassee, FL, United States; ^4^School of Biological Sciences, University of East Anglia, Norwich, United Kingdom

**Keywords:** dimethylsulfide, methanethiol, sulfur cycle, *mddA*, *mtoX*

## Abstract

Methanethiol (MeSH) and dimethylsulfide (DMS) are volatile organic sulfur compounds (VOSCs) with important roles in sulfur cycling, signaling and atmospheric chemistry. DMS can be produced from MeSH through a reaction mediated by the methyltransferase MddA. The *mddA* gene is present in terrestrial and marine metagenomes, being most abundant in soil environments. The substrate for MddA, MeSH, can also be oxidized by bacteria with the MeSH oxidase (MTO) enzyme, encoded by the *mtoX* gene, found in marine, freshwater and soil metagenomes. Methanethiol-dependent DMS production (Mdd) pathways have been shown to function in soil and marine sediments, but have not been characterized in detail in the latter environments. In addition, few molecular studies have been conducted on MeSH consumption in the environment. Here, we performed process measurements to confirm that Mdd-dependent and Mdd-independent MeSH consumption pathways are active in tested surface saltmarsh sediment when MeSH is available. We noted that appreciable natural Mdd-independent MeSH and DMS consumption processes masked Mdd activity. 16S rRNA gene amplicon sequencing and metagenomics data showed that *Methylophaga*, a bacterial genus known to catabolise DMS and MeSH, was enriched by the presence of MeSH. Moreover, some MeSH and/or DMS-degrading bacteria isolated from this marine environment lacked known DMS and/or MeSH cycling genes and can be used as model organisms to potentially identify novel genes in these pathways. Thus, we are likely vastly underestimating the abundance of MeSH and DMS degraders in these marine sediment environments. The future discovery and characterization of novel enzymes involved in MeSH and/or DMS cycling is essential to better assess the role and contribution of microbes to global organosulfur cycling.

## Introduction

Dimethylsulfide (DMS) is a volatile organic sulfur compound (VOSC) predominantly produced by marine bacteria through biotransformations of organosulfur compounds (Sievert et al., 2007; Curson et al., 2011). It is estimated that this process generates up to 33 × 10^12^ g S per year (Simó, 2010). However, only 10% of the DMS produced is likely released into the atmosphere, since most is further catabolised by bacteria or photochemically oxidized (Kiene and Bates, 1990). Despite this, DMS constitutes the most abundant biogenically-derived form of sulfur transferred from the sea to the atmosphere (Kiene and Bates, 1990). DMS oxidation products act as cloud condensation nuclei, aiding cloud formation over the oceans and affecting atmospheric chemistry (Sievert et al., 2007; Vallina and Simó, 2007). There is a significant transfer of sulfur to land when DMS or its oxidation products are delivered back to the Earth’s surface by precipitation. DMS is also a signaling molecule for some seabirds, crustaceans and marine mammals that use it as foraging cue (DeBose and Nevitt, 2008).

In marine environments DMS is predominantly produced from microbial catabolism of dimethylsulfoniopropionate (DMSP) via DMSP lyases enzymes (Curson et al., 2011; Johnston et al., 2016; Sun et al., 2016). However, there are DMS production pathways that are independent of DMSP and are not limited to marine environments, but these are generally thought to be minor contributors to global DMS production (Howard and Russell, 1996; Stets et al., 2004; Spielmeyer et al., 2011). Many anoxic environments such as freshwater lake sediments (Zinder and Brock, 1978; Lomans et al., 1997, 2001), saltmarsh sediments (Kiene and Visscher, 1987), cyanobacterial mats (Zinder et al., 1977) and peat bogs (Kiene and Hines, 1995) can produce DMS at similar levels to those described for the upper marine water column (Lomans et al., 1997; Lana et al., 2011), likely due to the microbial methylation of methanethiol (MeSH; Kiene and Hines, 1995; Stets et al., 2004). Carrión et al. (2015) showed that MeSH-dependent DMS production (Mdd) is common in aerobic bacteria through a S-adenosyl-Met-dependent methyltransferase MddA enzyme. Functional MddA enzymes are found in a wide range of bacteria including actinobacteria, rhizobiales, cyanobacteria and sediment-dwelling pseudomonads. The *mddA* gene is present in both marine and terrestrial metagenomes, but is much more abundant in soil environments, where it is predicted to occur in 5–76% of bacteria (Carrión et al., 2015). To study the prevalence of the Mdd pathway in the environment, Carrión et al. (2017) tested DMS production from MeSH in a wide range of soils, freshwater and marine samples. It was shown that tested soils produced higher concentrations of DMS in the presence of MeSH (up to 19.6 ± 0.6 nmol DMS per g of sample) than marine sediments (up to 6.4 ± 0.8 nmol DMS per g of sample). No DMS was detected in freshwater, seawater or beach sand samples after incubations with MeSH (Carrión et al., 2017). It should be noted that the above concentrations are a consequence of Mdd activity competing against MeSH and DMS degradation processes. Indeed, DMS produced in freshwater and marine environments is known to be rapidly metabolized by bacteria (Kiene and Visscher, 1987; Kiene and Bates, 1990; Lomans et al., 1997; Stets et al., 2004; Lyimo et al., 2009).

The substrate for Mdd, MeSH, is also a VOSC produced in marine environments from DMSP through the demethylation pathway (Howard et al., 2006) or as an intermediate of DMS degradation (Lomans et al., 1999, 2002; Bentley and Chasteen, 2004; Schäfer et al., 2010). Alternative sources of MeSH are the methylation of sulfide in anaerobic environments, degradation of sulfur-containing amino acids or demethiolation of sulfhydryl groups (Lomans et al., 2001, 2002; Bentley and Chasteen, 2004). Eyice et al. (2018) identified the *mtoX* gene encoding the MeSH oxidase (MTO) enzyme. MTO is a metalloenzyme that converts MeSH into formaldehyde, hydrogen sulfide and hydrogen peroxide. The MTO enzyme is found in sulfur-oxidizing and methylotrophic bacteria such as *Thiobacillus, Rhodococcus* and *Hyphomicrobium* strains (Suylen et al., 1987; Gould and Kanagawa, 1992; Kim et al., 2000; Lee et al., 2002; Eyice et al., 2018). Metagenomics analysis suggested that the *mtoX* gene is widely distributed in marine (0.4–45.6% of bacteria), freshwater (5.3% of bacteria) and soil environments (up to 6.3% of bacteria; Eyice et al., 2018). To our knowledge, no studies have been conducted to assess the activity of the MTO pathway in the environment, but Kiene (1996) and Kiene and Visscher (1987) have studied MeSH consumption in marine samples.

Here, we measure the activity of the Mdd pathway and consider MeSH and DMS consumption rates in surface saltmarsh sediment. This work represents an important step to evaluate the significance of the MTO and Mdd pathways in the environment. Finally, we combine culture-dependent and culture-independent methods to identify the microbes likely to be involved in DMS and MeSH cycling in this marine environment.

## Materials and Methods

### Sample Collection

Three oxic sediment samples were collected from the surface sediment layer (top 1–3 cm) of different ponds in Stiffkey saltmarsh (52°57′54.0″N, 0°55′31.0′E) using an acrylic corer in September 2015. Sampled ponds had a pH of 7.5–7.8, temperature of 14–15°C and salinity of 32–35 practical salinity units (PSU). Cores were transported immediately to the laboratory and processed on arrival.

### MeSH Consumption and DMS Production From MeSH by Saltmarsh Sediment Samples

To study MeSH degradation and DMS produced through the Mdd pathway, 1 g of saltmarsh sediment from each of the three biological samples was placed in a 125 ml sealed vial containing 20 ml distilled water 35 PSU, 5 % Marine Basal Medium (MBM; Baumann and Baumann, 1981), 10 mM mixed carbon source (C; 200 mM succinate, 200 mM glucose, 200 mM sucrose, 200 mM pyruvate, 200 mM glycerol) and 20 μmol MeSH added as sodium methanethiolate (Sigma-Aldrich). Additions of sodium methanethiolate are cited in text as additions of MeSH. Microcosm experiments were incubated at 22°C for 24 h before measuring headspace MeSH and DMS concentration by gas chromatography (GC) as detailed in Carrión et al. (2015). Sediment samples were autoclaved twice and used as controls to show that variations in MeSH and DMS concentrations in the headspace were due to biological activity.

### Saltmarsh Sediment Enrichments

**FIGURE 1:**
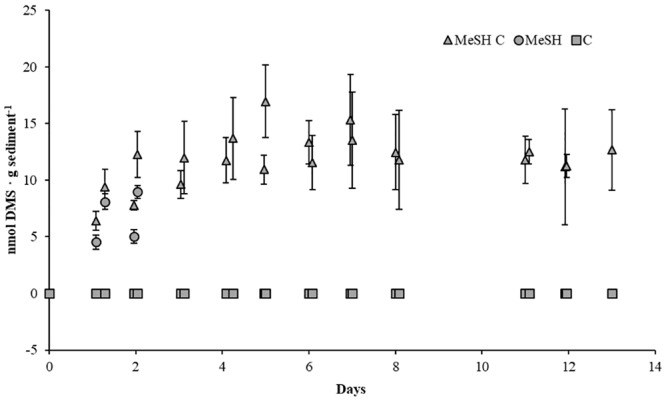
DMS concentrations in saltmarsh sediment enrichments. DMS concentration in the headspace of the sediment samples are expressed as nmol g soil^-1^. MeSH (20 μmol) was added daily to the sediment slurries with (MeSH C, triangles) and without a mixed carbon source (MeSH, circles) daily, as this gas was depleted after 24 h. No MeSH was added to the carbon-only control (C, squares). Values represent the average of three biological replicates with their respective standard deviations (smaller than marker if not visible).

To study the effects of MeSH on the processes of MeSH consumption and DMS production and consumption, as well as on bacterial diversity, three sets of enrichments were set up as above on each of the three biological samples. One set of enrichments was supplemented with mixed carbon source (C; 10 mM). The second set of microcosms was supplemented with MeSH (20 μmol). The third set of enrichments were supplemented with C (10 mM) and MeSH (20 μmol). Sterile controls (set up as above) were used to follow abiotic effects. Samples were incubated at 22°C for 14 days. Vials were opened daily to ensure oxic conditions during the incubation period and to amend MeSH-only and MeSH plus C samples with fresh MeSH as this gas disappeared after 24 h. MeSH and DMS concentrations in the headspace were monitored by GC (Figure 1).

### Isolation and Characterization of Strains

Samples from t0 and 14-day enrichments with C, MeSH and MeSH plus C were plated onto Marine Basal Medium (MBM) supplemented with C (10 mM), MeSH (20 μmol) or both, respectively. Colonies with different morphologies were inoculated into MBM and purity was checked by microscopy and plating onto Zobell Marine Broth medium (Buck and Cleverdon, 1960). Pure isolates were then tested for MeSH consumption and DMS production through the Mdd pathway.

To study DMS production and MeSH consumption, isolates were grown for 24 h at 30°C in MBM with C (10 mM) plus MeSH (0.3 μmol) or no substrate. Those isolates that consumed ≥ 95% MeSH and/or produced ≥ 50 pmol DMS mg prot^-1^ min^-1^ were further characterized and identified by sequencing their 16S rRNA genes with primers 27F and 1492R (Lane, 1991). At least one representative strain from each genus was selected to study DMS consumption, DMS production from DMSP, DMSP production and the use of MeSH and DMS as sole carbon sources.

To study DMS consumption, isolates were inoculated into serum vials containing MBM plus C (10 mM). Vials were supplemented with DMS (0.3 μmol; Sigma-Aldrich) and incubated at 30°C before monitoring DMS concentration in the headspace by GC.

To test the ability of isolates to cleave DMSP, bacterial strains were grown in serum vials with MBM plus C supplemented with DMSP (1 mM) for 24 h at 30°C before measuring DMS production by GC. Cultures with no substrate added were set up as controls. Isolates were tested for DMSP production as in Curson et al. (2017). Briefly, isolates were grown in MBM plus C for 24 h at 30°C. After this period, DMSP was subjected to alkaline lysis and DMS released from this reaction was quantified by GC.

The ability to use MeSH and DMS as sole carbon sources by representative strains of each genus was studied following the protocol described by Carrión et al. (2017). Briefly, isolates were grown overnight in MBM plus C. Cultures were pelleted and washed three times with fresh MBM containing no carbon source. Cultures where then adjusted to an OD_600_ of 0.6 and inoculated into fresh MBM containing no carbon source, C, MeSH or DMS at 2 mM concentration in airtight serum vials. After incubation at 30°C for 96 h, growth was estimated by measuring cell density at OD_600_.

Genomic DNA from bacterial isolates *Labrenzia* sp. mcm14, *Oceanicola* sp. mcm12, *Stappia* sp. mcm29 and *Rhodobacterales* bacterium cm12 was sequenced by Microbes NG (Birmingham, United Kingdom) using Illumina HiSeq technology. Resultant reads were assembled into contigs with SPAdes (Bankevich et al., 2012) and quality of the assemblies was assessed with QUAST (Gurevich et al., 2013).

### Rates of MeSH Consumption, DMS Production and DMS Consumption

Two sets of C (10 mM) plus MeSH (20 μmol) microcosm experiments were done to measure MeSH consumption and DMS production (set 1, in triplicate); and DMS consumption rates (set 2, with 9 replicates; Supplementary Figure S1). MeSH was added daily to both sets of microcosms for 13 (set 2) or 14 days (set 1; Supplementary Figure S1). At time 0, 7 and 14 days, one set of microcosms was supplemented with MeSH (20 μmol) to measure net MeSH consumption and DMS production rates by GC. At the same time points, DMS (0.5 μmol) was added to the other set of microcosms to estimate net DMS consumption rates (these were sacrificed after the DMS consumption rate measurement). Rates of DMS production and consumption were measured by GC and are expressed as nmol h^-1^ g sediment^-1^. Rates of MeSH consumption are expressed as μmol h^-1^g sediment^-1^. Rates of biological catabolism of MeSH and DMS were obtained after subtracting rates of chemical degradation. Abiotic MeSH and DMS degradation rates were estimated by measuring the disappearance of these gases in sterile controls.

### PCR and qPCR of *mtoX*

Bacterial strains that removed ≥ 95% MeSH present in the headspace were screened for the presence of the *mtoX* gene by PCR using primers (MtoX41Fmodv2inos and MTOX352Rmod) and conditions reported in Eyice et al. (2018).

Primers spanning ∼142 bp of the *mtoX* gene (202F1, 5′- GSNGAYGGNTAYGGNTAYG-3′ and 246R2, 5′-TTNCCRAANCKYTTCATNGCYTC-3′) were designed to perform qPCR on natural (t0) samples. This primer set was first tested on *mtoX* positive control strains *Ruegeria pomeroyi* DSS-3, *Hyphomicrobium* sp. VS and *Methylococcus capsulatus* Bath (Eyice et al., 2018). Non-MeSH-oxidizing strains *Pseudomonas deceptionensis* M1 and *Rhizobium leguminosarum* J391 soil bacteria that lack *mtoX* were used as negative control strains. Amplification products of the expected size (142 bp) were only obtained from genomic DNA from positive control strains. PCR products were sequenced after purification and confirmed as *mtoX* genes after bioinformatics analysis with BLASTx^1^.

*mtoX* qPCR assays were performed using a StepOnePlus instrument (Applied Biosystems) following the manufacturer’s instructions. Reactions (20 μl) contained 25–50 ng of DNA, 2.5 μM of each primer and 10 μl of SensiFast SYBR Hi-ROX kit (Bioline). qPCR reaction consisted of an initial denaturation step at 95°C for 3 min followed by 40 cycles of denaturation at 95°C for 20 s, primer annealing at 58°C for 20 s, extension at 72°C for 20 s and data acquisition at 83°C for 15 s to avoid quantification of primer dimers. Specificity of qPCR assays was determined from melting curves obtained by increasing the temperature 1°C per 30 s from 65°C to 90°C, followed by gel electrophoresis and clone library construction from qPCR products. Ten clones from natural (t0) saltmarsh sediment samples were obtained. Sequences had 80–96% identity at the derived amino acid level to MTO from ratified MeSH-oxidizers. No false positives were detected.

The copy number of *mtoX* genes was determined from qPCR of ten-fold dilution series (10^0^–10^9^ copies per μl) of DNA standards. Standards were prepared by cloning the *mtoX* gene of *Ruegeria pomeroyi* DSS-3 into the pGEM^®^T Easy vector (Promega) and using this as template DNA. The detection limit of the *mtoX* qPCR assay was 10^3^ copies per 20 μl reaction.

Absence of inhibitors in the qPCR reactions was confirmed by carrying out a qPCR assay with *mtoX* primers using 10-fold serial dilutions of environmental samples as in Farhan Ul Haque et al. (2018; Supplementary Figure S2).

Finally, *mtoX* copies were normalized to 16S rRNA gene copy number to estimate the abundance of MTO-containing microorganisms in saltmarsh sediment samples.

Number of copies of 16S rRNA genes was determined by qPCR using 519F and 907R primers (Lane, 1991). Reactions (20 μl) contained 0.3–0.5 ng DNA, 0.4 μM of each primer and 10 μl of SensiFast SYBR Hi-ROX kit. qPCR reaction consisted of an initial denaturation step at 95°C for 3 min, followed by 40 cycles of 95°C for 20 s, 55°C for 20 s and 72°C for 30 s. Data collection was performed at 72°C for 15 s. Specificity of the qPCR reaction and quantification of 16S rRNA gene copy number were determined as above.

### 16S rRNA Gene Amplicon Sequencing

DNA was extracted from three biological replicates from t0 samples and enrichments with C, MeSH and MeSH plus C as described in Carrión et al. (2017). Extracted DNA was subjected to 16S rRNA gene amplicon sequencing by MR DNA (Shallowater, TX, United States), obtaining an average of 85,975 reads per sample with an average length of 300 bp. Operational taxonomic units (OTUs) were defined by clustering at 3% divergency. Taxonomy of the OTUs was assigned using BLASTn against a curated database derived from RPDII^2^ and NCBI (see footnote 1).

### Metagenomic Analysis of DNA From Saltmarsh Sediment Samples

Metagenomic analysis was performed by combining in equal proportions DNA extracted from biological replicates from t0 samples and 14-day enrichments with C, MeSH and MeSH plus C. Libraries were prepared as in Carrión et al. (2017), obtaining an average library size of 1,470 bp for t0 samples, 1,417 bp for samples enriched with C, 1,628 bp, for samples enriched with MeSH and 574 bp or samples enriched with MeSH plus C. Reads were quality-filtered and trimmed using Trimmomatic (Bolger et al., 2014), obtaining an average of 15,462,338 reads per sample with an average length of 151 bp. Metagenomes were then assembled using SPAdes assembler with kmers 55 to 127 (Bankevich et al., 2012), and assemblies were analyzed using Quast (Gurevich et al., 2013). N50 values were ∼1 kb for all metagenomes assemblies.

The abundance of functional genes in unassembled metagenomes was determined by tBLASTx (see footnote 1) of selected ratified gene sequences (*mddA, ddhA, dmoA, tmm, megL*, and *mtoX*) against the raw reads (E ≤ e^-5^). Each potential MddA, DdhA, DmoA, Tmm sequence retrieved from the analysis of metagenomes was manually checked by BLASTp against the RefSeq database. Any sequences with < 40% amino acid identity to ratified sequences detailed in Supplementary Table S1 were discarded. Only unique hits were counted. Hit numbers were normalized against read number of the smallest sample, to gene length and to hits of *recA*. Phylogeny of *mddA* and *mtoX* unique hits was analyzed using QIIME (Caporaso et al., 2010; MacQIIME version 1.9.0) by mapping the reads to a hand-curated reference database of 176 full-length *mddA* sequences and 500 *mtoX* sequences, using the blat method (Kent, 2002) for OTU picking and a cut-off of 45% amino acid identity. Taxonomy of unassembled metagenomes was further analyzed using MetaPhlAn (Segata et al., 2012; version 2.2.0).

To study the diversity of *mddA* and *mtoX* genes in the assembled metagenomes, contigs containing *mddA* and *mtoX* sequences were identified using tBLASTx (see footnote 1) and a cut-off of E ≤ e^-5^ to ratified MddA and MTO proteins. The phylogenetic tree was then constructed from *mddA* and *mtoX* sequence data using the ARB software package (Ludwig et al., 2004; version 6.0.1). Metagenomics contigs with hits to *mddA* and *mtoX* were aligned to a hand-curated reference database of 176 full-length *mddA* sequences and 36 full-length *mtoX* sequences, respectively. Contig sequences that could not be sufficiently aligned were discarded. *mddA* and *mtoX* RAxML phylogenetic trees (Stamatakis, 2014; version 8) were calculated using Maximum Likelihood on protein level with 100 replicates.

### Statistical Analysis

Statistical analyses were performed in R 3.3.2 (R Core Team, 2016) using the base statistics package, except where otherwise stated. The compositions package (Van den Boogaart and Tolosana-Delgado, 2008) was used for appropriate transformation and assessment of the effect of treatments on microbial composition data. Prior to multivariate analyses, each dataset (Class and Genus-level data) was transformed using a centered log-ratio (clr) transformation. Microbial community response was then assessed as follows.

Due to violation of traditional MANOVA assumptions, a non-parametric (permutational) MANOVA (McArdle and Anderson, 2001) was used, as implemented by the adonis2 function in the “vegan” package (Oksanen et al., 2017) with sediment treatment (4 levels) as the sole explanatory factor. Within each dataset 10,000 random permutations of treatment assignments were used to generate a null distribution, followed by permutation significance tests with pseudo-F ratios. To guard against confounding effects of location and dispersion, PCA plots (first two principle components) were visually examined and explicitly tested for differences in dispersion using betadisper and permutes functions in the “vegan” package. Higher replicate-replicate difference was consistently found within the “C” treatment, so the multivariate analyses were subsequently re-run leaving each of these replicates out in turn. This sequential omission did not alter the outcome of the analysis in any substantial way. Therefore, only the results from the full dataset are presented here. Linear Discriminant Analysis (LDA), using the MASS package (Venables and Ripley, 2002) lda function served as a *post hoc* test of multivariate treatment differences.

Univariate (taxon by taxon) percentage responses to treatments were analyzed on the same clr-transformed scale by ANOVA, with *p*-values adjusted (Benjamini-Hochberg correction; Benjamini and Hochberg, 1995) for multiple comparisons. For every significant univariate response thus determined, Tukey Honest Significant Difference (HSD) tests (95% family-wide confidence levels) were applied to determine *post hoc* pair-wise differences between treatments.

## Results

### MeSH Consumption and DMS Production From MeSH by Saltmarsh Sediment Samples

To assess the prevalence of the Mdd pathway in marine environments, surface sediment samples from Stiffkey saltmarsh were incubated in the presence and absence of MeSH. Unamended samples produced no VOSCs at detectable levels, suggesting that either DMS and MeSH are not abundant in this saltmarsh sediment or more likely, that these gases are quickly consumed by the microbial population as suggested by processes rates measured in this study (see below). Samples supplemented with MeSH (20 μmol) consumed it all in 24 h and produced similar levels of DMS to those previously reported for this environment (7.2 ± 0.6 nmol g sediment^-1^; Carrión et al., 2017). In addition, sediment samples consumed 44.4 ± 4.2% of the DMS added (0.5 μmol) after 24 h of incubation. These results confirm that MeSH is efficiently consumed by microbes and that Mdd and, likely, MeSH oxidation pathways in Stiffkey saltmarsh sediment are active when MeSH is available. Microbial DMS consumption processes were also active in this environment potentially hiding natural DMS production that was below our detection limits of 0.15 nmol for DMS and 4 nmol for MeSH.

### Saltmarsh Sediment Enrichments With MeSH

To study how the microbial diversity and DMS production rates in the saltmarsh sediment changed in response to the presence of MeSH and carbon availability, three sets of microcosm experiments were set up (see section “Materials and Methods”). Microcosms amended with C showed no MeSH or DMS production above the detection limit (Figure 1), confirming the requirement for MeSH in this marine environment for the Mdd pathway to produce detectable amounts of DMS. Samples supplemented with MeSH showed increasing amounts of DMS production over the first 2 days, reaching a maximum of 9.0 ± 0.7 nmol DMS g sediment^-1^. After this period, MeSH was still consumed every 24 h, but no DMS production was observed (Figure 1). Finally, microcosms enriched with MeSH plus C showed progressive increases in DMS production over the first 7 days up to 16.9 ± 3.2 nmol DMS g sediment^-1^. After that, DMS production steadily declined to 12.7 ± 3.6 nmol DMS g sediment^-1^ after 14 days (Figure 1). Therefore, higher DMS production levels (∼2.2-fold) were seen when MeSH was added in the presence of an additional carbon source. This is expected since a higher proportion of MeSH will likely be assimilated in the absence of other added carbon sources, especially when the sediment carbon reservoirs are exhausted.

### MeSH Consumption, DMS Production and DMS Consumption Rates

MeSH consumption and DMS production and consumption rates were estimated in saltmarsh sediment samples enriched with MeSH plus C since they showed the highest Mdd activity (see above).

DMS production rates appeared to decrease with the incubation time of the microcosm experiments (Supplementary Figure S3). The DMS produced 1–2 h after the addition of MeSH declined from 8.5 ± 0.2 nmol DMS g sediment^-1^ in t0 samples to 3.5 ± 0.9 nmol DMS g sediment^-1^ in the 7-day enrichments and to 3.1 ± 0.5 nmol DMS g sediment^-1^ after 14 days. After these initial increases in Mdd production (1–2 h), DMS concentration in the samples steadily decreased (Supplementary Figure S3) due to higher MeSH and DMS consumption rates (see below). No DMS was detected in the sterile sediment controls, indicating that measured DMS is solely due to biological activity.

The levels of both DMS and MeSH decreased in the sterile sediment controls, potentially indicating the chemical oxidation of these gases to dimethyldisulfide and dimethylsulfoxide, respectively (Kiene, 1996; Hatton, 2002; Bentley and Chasteen, 2004). However, biological removal always surpassed those levels in the sterile controls (Tables 1, 2).

**Table 1:** DMS consumption rates in saltmarsh sediment enrichments with MeSH plus C.

Day	Total DMS consumption	Abiotic DMS consumption	Biological DMS consumption
0	4.7 ± 0.3	1.3 ± 0.6	3.3 ± 0.3
7	7.4 ± 0.9	2.1 ± 0.7	5.3 ± 0.9
14	18.4 ± 0.7	2.3 ± 0.4	16.1 ± 0.7

*Rates of biological consumption of DMS were obtained after subtracting the abiotic DMS consumption from the total DMS consumption. Abiotic DMS consumption rates were estimated by measuring the disappearance of this gas in sterile sediment controls. Rates are expressed as nmol DMS g sediment^-*1*^ h^-*1*^.*

**Table 2:** MeSH consumption rates in saltmarsh sediment enrichments with MeSH plus C.

Day	Total MeSH consumption	Abiotic MeSH consumption	Biological MeSH consumption
0	9.5 ± 0.3	3.2 ± 0.6	6.3 ± 0.4
7	15.1 ± 0.2	6.6 ± < 0.1	8.4 ± 0.2
14	21.8 ± < 0.1	9.1 ± < 0.1	12.6 ± < 0.1

*Rates of biological consumption of MeSH were obtained after subtracting the abiotic MeSH consumption from the total MeSH consumption. Abiotic MeSH degradation rates were estimated by measuring the disappearance of this gas in sterile sediment controls. Rates are expressed as μmol MeSH g sediment^-*1*^h^-*1*^.*

Biological DMS consumption rates progressively increased from t0 (3.3 ± 0.3 nmol DMS g sediment^-1^h^-1^) to 7 days (5.3 ± 0.9 nmol DMS g sediment^-1^h^-1^) to reach a maximum of 16.1 ± 0.7 nmol DMS g sediment^-1^h^-1^ after 14 days of incubation (Table 1). These increasing rates with time likely explain the reduction in DMS production seen throughout the incubations with MeSH plus C.

Biological MeSH consumption rates also increased from t0 (6.3 ± 0.4 μmol MeSH g sediment^-1^h^-1^) to 7 days (8.4 ± 0.2 μmol MeSH g sediment^-1^h^-1^), with the maximum rate being observed after 14 days of incubation (12.6 ±≤ 0.1 μmol MeSH g sediment^-1^h^-1^; Table 2).

These data indicate that the extended exposure of the surface saltmarsh sediment to MeSH enhanced both MeSH and DMS consumption. It is also likely that the Mdd pathway is enhanced by the presence of MeSH but this is not directly detected due to the MeSH substrate and DMS product being consumed at a greater overall rates, by an Mdd-independent MeSH degradation process in the case of the former. Such increased rates may be due to an enrichment of bacteria with an active Mdd pathway, termed Mdd^+^ (either contain *mddA* or undetermined genes with equivalent function) and/or MeSH and DMS-degrading microorganisms from this saltmarsh environment.

### Surface Saltmarsh Microbial Community Changes in Response to MeSH Exposure

**FIGURE 2:**
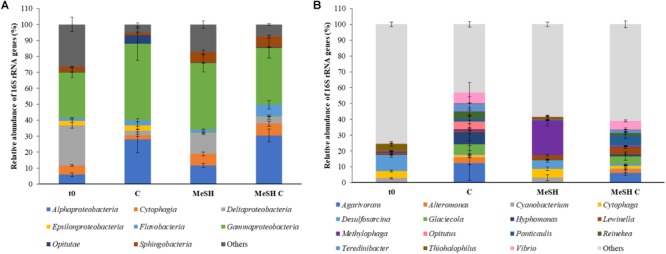
Taxonomic profiling of the 16S rRNA gene amplicon sequencing data from saltmarsh sediment enrichments. **(A)** Class level; **(B)** Genus level. Only classes or genera that are ≥ 5% abundant in at least one of the conditions are represented. t0: natural samples; C: enrichments with mixed carbon source; MeSH: samples enriched with MeSH-only; MeSH C: enrichments with MeSH plus C. Values represent the average of three biological replicates with their respective standard deviations. Results for the individual replicates are shown in Supplementary Figure S4.

To study the microbial community changes caused by the addition of MeSH, DNA from t0 samples and enrichments with C, MeSH and MeSH plus C was subjected to 16S rRNA gene amplicon sequencing and metagenomics. Amplicon sequencing data showed that the natural (t0) bacterial population was dominated by microorganisms belonging to *Gammaproteobacteria* (28.4 ± 3.1%) and *Deltaproteobacteria* (25.2 ± 1.8%, Figure 2) classes. At the genus level, members of *Desulfosarcina* (10.1 ± 1.3%), *Thiohalophilus* (4.4 ± 1.5%) and *Cytophaga* (4.3 ± 0.6%) were most abundant in t0 saltmarsh sediment samples (Figure 2). All incubation experiments after 14 days had vastly different profiles to that of the microbial community of the natural sediment (Figure 2).

In C-only enrichments, *Alphaproteobacteria* showed a significant increase in relative abundance compared to t0 samples (up to 27.9 ± 8.3%, *p* < 0.001, Tukey honest significant difference, HSD), whereas *Deltaproteobacteria* were drastically reduced to 3.0 ± 0.9% (*p* < 0.001, Tukey HSD). The microbial composition between biological replicates showed considerable differences at the genus level (Supplementary Figure S4). For example, the most abundant genera in replica one were *Glacieola* (13.8%) and *Hyphomonas* (13.2%), whereas replica two was dominated by *Agarivorans* (24.3%) and *Vibrio* (13.6%), and *Agarivorans* (10.2%) and *Alteromonas* (8.5%) were most abundant in replica three. Nevertheless, in all microcosms amended with C, these genera were more abundant than in t0 samples (Supplementary Figure S4).

The *Cytophagia* and *Sphingobacteria* classes significantly increased their relative abundance in the MeSH-only incubations up to 7.1 ± 1.2% (*p* = 0.010, Tukey HSD) and 6.6 ± 1.5% (*p* = 0.001, Tukey HSD), respectively, versus the t0 samples. At a genus level, the extended exposure to MeSH resulted in *Methylophaga* being the most abundant genus in these samples (21.9 ± 3.5%, *p* = 0.021, Tukey HSD; Figure 2). This is also supported by the metagenomic analysis which showed that *Methylophaga* was highly enriched (to 63.3%) in the MeSH-only incubations compared to t0 samples (Supplementary Figure S5). *Methylophaga* contains species capable of one carbon metabolism, with some containing the MTO enzyme and catabolising both MeSH and DMS (De Zwart et al., 1996; Kim et al., 2007; Schäfer, 2007; Neufeld et al., 2008; Boden et al., 2010; Eyice et al., 2018). Thus, it is not surprising to see this genus enriched in the samples amended with MeSH.

In the incubations with MeSH plus C, *Alphaproteobacteria* (30.5 ± 4.0%, *p* < 0.001, Tukey HSD), *Cytophagia* (7.4 ± 1.6%, *p* = 0.032, Tukey HSD), *Flavobacteria* (7.5 ± 2.6%, *p* < 0.001, Tukey HSD) and *Sphingobacteria* (6.7 ± 0.8, *p* = 0.003, Tukey HSD) were significantly more abundant compared to t0 samples, whereas *Deltaproteobacteria* were significantly reduced (to 4.1 ± 1.3%, *p* < 0.001, Tukey HSD). At the genus level, MeSH plus C enrichments showed significant increases in the relative abundance of *Agarivorans* (up to 6.1 ± 1.6%, *p* = 0.016, Tukey HSD) and *Ponticaulis* (up to 6.6 ± 1.9%, *p* = 0.010, Tukey HSD), but a decrease in *Desulfosarcina* (to 1.1 ± 0.2%, *p* < 0.001, Tukey HSD) compared to the t0 samples (Figure 2). Metagenomic data also showed that *Ponticaulis* was the most abundant genus (58.7%) in the enrichments with MeSH plus C (Supplementary Figure S5).

### Abundance of Genes Encoding Enzymes Involved in MeSH and DMS Metabolism

Metagenomic data were also screened for the presence and relative abundance of key genes involved in MeSH and DMS metabolism (see Methods). These genes included *megL* encoding methionine gamma lyase that cleaves Met to MeSH (Tanaka et al., 1976), *mddA, mtoX*, the DMS dehydrogenase gene *ddhA* (McDevitt et al., 2002), the DMS monooxygenase gene *dm*oA Boden et al., 2011) and the trimethylamine monooxygenase gene *tmm* (Lidbury et al., 2016).

Metagenomic analysis indicated that *megL* was very abundant in the saltmarsh sediment natural (t0) microbial community (78.4% of bacteria), indicating the high potential of the bacterial population to generate MeSH from Met. Furthermore, 9.6% of bacteria from t0 samples contained *mddA* and 4.0 % contained the MeSH oxidase gene *mtoX*. This relatively high *mtoX* abundance in the sediment saltmarsh samples was confirmed by qPCR assays, which estimated 0.05 ± 0.01 *mtoX* genes per copy of 16S rRNA gene. *ddhA* was the most abundant gene involved in DMS degradation in the t0 community (13.3% of bacteria), followed by *tmm* (2.1% of bacteria) and *dmoA* (0.5% of bacteria; Table 3). Given the predicted number of bacterial cells in Stiffkey surface sediment (∼2 × 10^10^ ± 1.5 × 10^8^; Williams et al., unpublished), these values suggest a potentially huge microbial biomass with the genetic potential to cycle DMS and MeSH in these environments. This large potential biomass together with the observed processes rates, above, suggest that Mdd and DMS degradation are likely to be important under certain conditions in this marine environment.

**Table 3:** Comparison of normalized values of functional genes of interest in saltmarsh sediment unassembled metagenomes.

Sample	MegL	MddA	MTO	DdhA	DmoA	Tmm
t0	78.4	9.6	4.0	13.3	0.5	2.1
C	76.9	13.2	0.1	3.4	0.2	2.8
MeSH C	60.1	5.9	0.5	2.9	0.2	2.3
MeSH	84.5	9.8	24.2	8.5	0.3	2.8

*Unique hits of target genes were normalized to the read number of the smallest sample, to gene length and to unique hits of *recA* to predict the percentage of bacteria that contain the target genes. t0’ natural samples; C’ enrichments with mixed carbon source after 14 days; MeSH C’ samples incubated with mixed carbon source and MeSH for 14 days; MeSH’ samples enriched with MeSH for 14 days.*

The percentage of bacteria containing *mddA* remained constant under all the enrichment conditions, except for those samples enriched with MeSH plus C after 14 days, in which its relative abundance decreased to 5.9% (Table 3). This is in accordance with the decreased initial DMS production levels observed when the MeSH was added to the microcosms experiments (Supplementary Figure S3). These same enrichments showed increased MeSH consumption rates after 14 days, yet the relative abundance of *mtoX* decreased (∼ 8-fold) to 0.5%. These data are consistent with the MeSH plus C incubations enriching for bacteria containing novel Mdd-independent MeSH-degrading enzymes, e.g., MeSH-oxidizing enzymes (see below).

Only the MeSH-alone incubations showed an increase in the abundance of bacteria predicted to contain *mtoX* (up to 24.2%) compared to the t0 samples. As detailed above, this was expected since members of *Methylophaga* were heavily enriched in these samples and bacteria of this genus contain *mtoX* and oxidise MeSH and DMS (De Zwart et al., 1996; Kim et al., 2007; Schäfer, 2007; Neufeld et al., 2008; Boden et al., 2010; Eyice et al., 2018).

Interestingly, the MeSH plus C enrichments, which had vastly increased DMS consumption rates (5-fold) compared to t0, actually showed a ∼5-fold reduction in abundance (to 2.9%) of the DMS dehydrogenase gene *ddhA*. Furthermore, the relative abundance of other known DMS-degrading genes (*dmoA* and *tmm*) showed only slight variations in abundance between the tested conditions (Table 3). These data, together with the culture-dependent work (see below), suggest there might be bacteria with novel DMS-cycling enzymes in Stiffkey saltmarsh surface sediment.

### Diversity of *mddA* and *mtoX* in Saltmarsh Sediments Metagenomes

**FIGURE 3:**
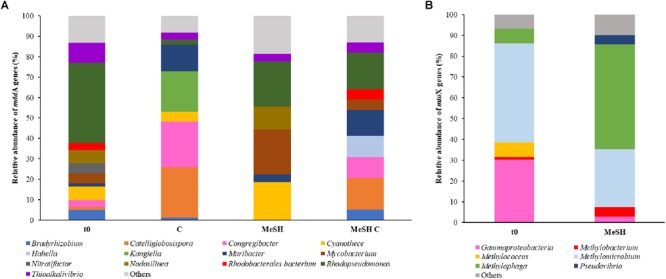
Diversity and relative abundance of *mddA* and *mtoX* genes in saltmarsh sediment unassembled metagenomes. **(A)** Only *mddA* genes with abundance of ≥ 5% in at least one of the conditions are represented. **(B)** Only *mtoX* genes that are at least ≥ 4% abundant in one condition are shown. C and MeSH plus C metagenomes are not represented as they yielded < 10 *mtoX* unique hits. t0: natural samples; C: enrichments with mixed carbon source; MeSH: samples enriched with MeSH-only; MeSH C: enrichments with MeSH plus C.

Metagenomes from saltmarsh sediment samples were also analyzed to study changes in the diversity of *mddA* and *mtoX* genes after the exposure to MeSH. Analysis of the unassembled metagenomes revealed that the predominant *mddA* sequences in the natural (t0) bacterial population (39.3%) were closely related to *mddA* from *Rhodopseudomonas* (Figure 3), although sequences similar to *mddA* from *Thioalkalivibrio* (9.8%), *Nodolisinea* (6.6%), *Cyanothece* (6.6%) and *Mycobacterium* (4.9%) were also present (Figure 3).

In the MeSH incubations *Rhodopseudomonas mddA* was still abundant but decreased to represent 22.2% of the sequences, whereas *Cyanothece* and *Mycobacterium mddA* increased in relative abundance (to 18.5 and 22.2%, respectively) compared to t0. Samples incubated with C also showed decreased *Rhodopseudomonas mddA* relative abundance (2.1%), but increases in *mddA* genes from *Catelliglobosispira* (24.7%), *Congregibacter* (22.4%), *Kangiella* (20%) and *Maribacter* (13%). Finally, in the MeSH plus C enrichments, *mddA* sequences phylogenetically related to *mddA* from *Rhodopseudomonas* were the most abundant *mddA* genes in the bacterial population (17.9%), followed by *Catellioglobosispira* (15.4%) and *Maribacter* (12.8%) *mddA* homologs. These data indicate that the diversity of *mddA* genes is affected by the different enrichment conditions.

**FIGURE 4:**
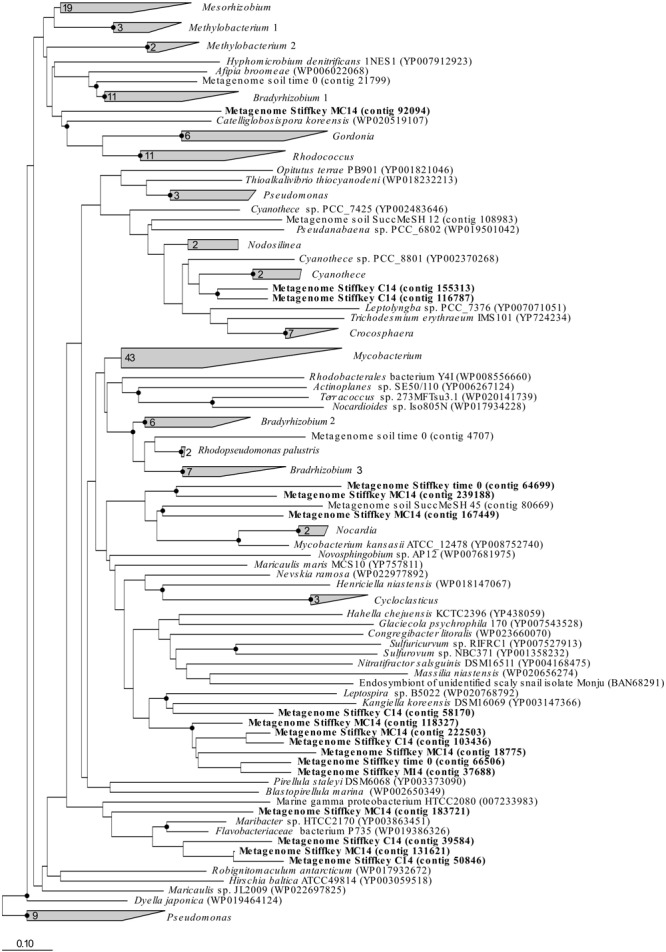
*mddA* RAxML phylogenetic tree including sequences retrieved from saltmarsh sediment assembled metagenomes. Environmental sequences obtained in this study are marked in bold. Time 0: sequences obtained from natural samples; M14: sequences retrieved from MeSH enrichments after 14 days of incubation; C14: sequences yielded by 14-day enriched samples with mixed carbon source; MC14: sequences obtained from enrichments with MeSH plus C after 14 days of incubation. Bar, 0.10 substitutions per amino acid position. Bootstrap values ≥ 70% (based on 100 replicates) are represented with dots at branch points.

The analysis of the assembled saltmarsh sediment metagenomes confirmed the presence of sequences similar to *mddA* genes from *Mycobacterium* (in t0 samples), *Kangiella* (in C-only incubations), *Maribacter* (in C-only and MeSH plus C samples) and *Catellioglobosispora* (in MeSH plus C enrichments) common to the unassembled metagenomics data (Figure 4). However, *mddA* sequences related to *Kangiella* (in t0, MeSH-only and MeSH plus C samples), *Cyanothece* (in C-only enrichments) and *Mycobacterium* (in MeSH plus C incubations) were detected in the assembled metagenomes but had a low relative abundance in the unassembled metagenomes. These discrepancies can potentially be explained by the fact that the analysis of the assembled metagenomes do not always give results wholly representational of the original taxonomy of the sample due to the bioinformatics challenges posed by complex metagenomic communities, such as non-uniform read coverage and sequences similarity/diversity between closely related species (Nayfach and Pollard, 2016; Vollmers et al., 2017).

**FIGURE 5:**
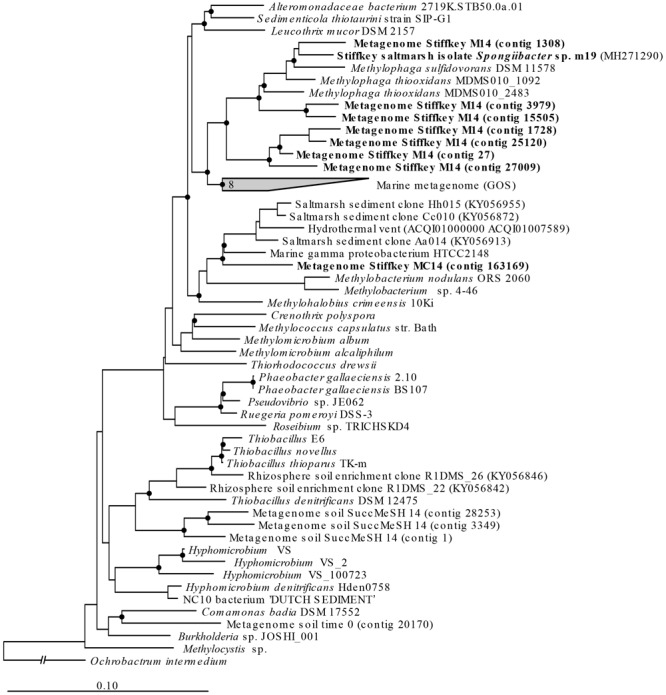
*mtoX* RAxML phylogenetic tree including the sequences retrieved from saltmarsh sediment assembled metagenomes. Environmental sequences obtained in this study are marked in bold. Time 0: sequences obtained from natural samples; M14: sequences retrieved from MeSH enrichments after 14 days of incubation; C14: sequences yielded by 14-day enriched samples with mixed carbon source; MC14: sequences obtained from enrichments with MeSH plus C after 14 days of incubation. Bar, 0.10 substitutions per amino acid position. Bootstrap values ≥ 75% (based on 100 replicates) are represented with dots at branch points.

Only metagenomes from t0 samples and the MeSH-only enrichments were used to study the changes in the *mtoX* gene diversity since the C or MeSH plus C incubations yielded < 10 *mtoX* unique hits. Analysis of t0 samples showed that the dominant *mtoX* sequences in the natural bacterial population were closely related to *Methylomicrobium* (47.7% of *mtoX* genes) and unidentified Gammaproteobacteria (30.3%). As expected, the relative abundance of *mtoX* genes similar to *mtoX* from *Methylophaga* dramatically increased up to 50.5% in the MeSH-only enrichments, whereas *mtoX* genes from *Methylobacterium* and unidentified Gammaproteobacteria were less abundant (28.0 and 2.9%, respectively; Figure 3). The analysis of the saltmarsh sediment assembled metagenomes also confirmed that the *mtoX* sequences retrieved from samples enriched with MeSH-only were phylogenetically related to *mtoX* from *Methylophaga* (Figure 5).

### Isolation and Characterization of Strains

Cultivation-dependent methods were used to isolate and study bacteria from saltmarsh surface sediment specifically looking at their ability to consume MeSH and/or produce DMS through the Mdd pathway. Isolates were obtained from t0 samples (37) and from 14-day enriched samples with C (62), MeSH (43) or MeSH plus C (71).

35.1% of isolates obtained from t0 samples consumed ≥ 75% of 0.3 μmol MeSH added to the headspace, but only 13.5% were Mdd^+^ producing > 10 pmol DMS mg protein^-1^min^-1^ from MeSH. The percentage of isolates that produced DMS above 10 pmol DMS mg protein^-1^min^-1^ increased in both the samples enriched with MeSH plus C (to 22.9%) and C (to 25%), whereas slightly decreased in the MeSH-only enrichments (to 10.6%). This was expected since a higher proportion of MeSH would probably be assimilated for growth in the absence of other carbon sources, especially after 14 days incubation when the sediment carbon reserves are likely depleted. Conversely, the percentage of isolates able to degrade ≥ 75% of MeSH increased up to 38.2% in the MeSH- only enrichments and decreased to 17.1% in the samples amended with MeSH plus C. The C incubations had the lowest percentage of MeSH-degrading isolates (4.7%), suggesting that the presence of excess carbon favors bacteria lacking this ability or that cultivable bacteria from incubations including C generally may have lower MeSH-degrading activity.

Isolates that either consumed ≥ 95% of the MeSH present in the headspace or produced ≥ 50 pmol DMS mg protein^-1^min^-1^ were identified by sequencing their 16S rRNA genes. At least one representative of each genus (Supplementary Figure S6) was selected for further characterization.

Isolates that degraded ≥ 95% of MeSH present in the headspace, were mainly *Gammaproteobacteria* (*Microbulbifer, Halomonas, Vibrio, Spongiibacter* and *Pseudomonas* sp.) and *Alphaproteobacteria* strains (*Oceanicola, Labrenzia, Stappia, Rhododobacter* and a *Rhodobacterales* bacterium; Table 4 and Supplementary Figure S6). However, only one of these, *Spongiibacter* sp. m19, was found to contain *mtoX* when their genomes were screened by PCR using *mtoX* degenerate primers (see Methods). The *Spongiibacter* sp. m19 *mtoX* showed 89% identity at the derived amino acid level to MTO from *Methylophaga thiooxydans* DMS010, a *bona-fide* MeSH-oxidizing microorganism (Eyice et al., 2018). Interestingly, 72.7% of the isolates that metabolized MeSH were also able to degrade DMS (Table 4), but none of them could use MeSH or DMS as sole carbon sources (data not shown). Also, 72.7% of the isolates that consumed MeSH were able to generate DMS from DMSP, but not from MeSH, indicating they likely have a DMSP lyase enzyme and that the major MeSH consumers may not be Mdd^+^ bacteria. The only exceptions were *Vibrio* sp. m18 and *Rhodobacterales* bacterium cm12, which produced DMS from MeSH, although at low levels (<10 pmol DMS mg protein^-1^min^-1^; Table 4). No sequenced *Vibrio* strains contain MddA and the sequenced genome of *Rhodobacterales* bacterium cm12 also lacked this gene, suggesting they both utilize a novel DMS-producing pathway.

**Table 4:** Characterization of MeSH, DMS and DMSP metabolism of bacterial isolates obtained in this study.

Strain	MeSH consumption	DMS consumption	DMS from MeSH	DMS from DMSP	DMSP
*Microbulbifer* sp. t0_20	100 ± < 0.1	ND	ND	372.3 ± 49.7	ND
*Oceanicola* sp. mcm12	100 ± < 0.1	98.9 ± 1.8	ND	26.5 ± 7.1	13.9 ± 0.1
*Labrenzia* sp. mcm14	98.7 ± 1.8	100 < 0.1	ND	9.1 ± 3.4	12.1 ± 0.9
*Stappia* sp. mcm29	100 ± < 0.1	99.0 ± 1.8	ND	1.3 ± 0.7	9.3 ± 0.3
*Halomonas* sp. m15	100 ± < 0.1	ND	ND	1.2 ± 0.1	ND
*Vibrio* sp. m18	99.3 ± 1.2	ND	2.4 ± 0.4	1.5 ± 0.1	ND
*Spongiibacter* sp. m19	100 ± < 0.1	100 ± < 0.1	ND	ND	ND
*Rhodobacter* sp. mm8	100 ± < 0.1	100 ± < 0.1	ND	ND	ND
*Pseudomonas* sp. mm10	100 ± < 0.1	100 ± < 0.1	ND	1391.0 ± 849.9	ND
*Rhodobacterales* bacterium cm12	100 ± < 0.1	100 ± < 0.1	9.8 ± 2.0	13.5 ± 5.9	12.2 ± 0.5
*Alteromonas* sp. cm21	96.5 ± 0.5	53.0 ± 4.5	ND	ND	ND
*Marinobacter* sp. t0_31	NT	NT	91.2 ± 32.6	881.1 ± 58.3	ND
*Hyphomonas* sp. t0_m34	NT	NT	110.1 ± 8.2	5.4 ± 1.6	ND
*Vibrio* sp. mc9	NT	NT	173.4 ± 8.2	22.2 ± 2.6	5.1 ± 0.2
*Alteromonas* sp. cm34	NT	NT	324.3 ± 113.8	ND	ND

*MeSH and DMS consumption is expressed as percentage of gas removed from the headspace compared to t0. DMS and DMSP production rates are expressed as pmol mg protein^-*1*^ min^-*1*^. Results shown are the average of three biological replicates with their respective standard deviations. ND’ not detected; NT’ not tested.*

The genomes of MeSH and DMS-degrading strains *Labrenzia* sp. mcm14, *Oceanicola* sp. mcm12, *Stappia* sp. mcm29 and *Rhodobacterales* bacterium cm12 were sequenced. All of these bacteria except the Mdd^+^ cm12 strain contained proteins with 76% amino acid identity to the DMS dehydrogenase DdhA from *Rhodovulum sulfidophilum*, which oxidizes DMS to DMSO for energy and not for carbon utilization (McDevitt et al., 2002). This would explain how these microorganisms degrade DMS and perhaps why they cannot use it as a sole carbon source. Moreover, none of the strains contained homologs to MTO, suggesting that they might possess novel enzymes involved in MeSH catabolism.

Isolates that produced ≥ 50 pmol DMS mg protein^-1^min^-1^ from MeSH were *Alpha-* and *Gammaproteobacteria* strains belonging to *Marinobacter, Hyphomonas* and *Alteromonas* genera (Table 4 and Supplementary Figure S6). No genome-sequenced bacteria from these genera contain MddA. Thus, we do not understand the mechanisms of DMS production in these isolates. When incubated with DMSP all these strains except for *Alteromonas* sp. cm34 produced DMS (Table 4), indicating that they probably harbor DMSP lyases genes.

When the isolates were screened for DMSP production, *Oceanicola* sp. mcm12, *Labrenzia* sp. mcm14, *Rhodobacterales* bacterium cm12, *Stappia* sp. mcm29 and *Vibrio* sp. mc9 were found to accumulate between 5.1 and 13.9 pmol mg protein^-1^ min^-1^ DMSP (Table 4), which may act as a source of DMS in the natural environment. This is the first report of a *Vibrio* species producing DMSP.

## Discussion

This study assesses the importance of the Mdd pathway and marine VOSCs cycling in surface marine sediment from Stiffkey saltmarsh. In these natural surface sediment samples DMS production was below the detection limit, but as with other tested sediment environments (Carrión et al., 2017), DMS production was stimulated by the addition of MeSH. In contrast, the natural sediment had significant initial MeSH and DMS consumption rates suggesting that these gasses are constantly cycling in this environment. Further analysis of the sediment through incubation experiments showed that the already sizeable natural MeSH and DMS turnover rates increased more so with extended MeSH incubation (up to 2 and 5-fold, respectively after 14 days). Both microbial DMS and MeSH catabolic processes affected the amount of DMS generated by Mdd^+^ microorganisms, through reducing the available MeSH substrate and DMS product, respectively. These findings support the hypothesis that the Mdd pathway is active in these natural saltmarsh sediments, but that the process has a limited effect on DMS transfer to the overlying water/atmosphere because of the high microbial sediment turnover rates of DMS and MeSH (a combination of Mdd and degradation pathways). Metagenomics analysis and culture-dependent work supports the above hypothesis by revealing that genes involved in DMS production (*mddA*) and consumption (*dmoA, ddhA* and *tmm*) as well as MeSH oxidation (*mtoX*) and bacteria that carry out these processes are abundant in the natural saltmarsh sediment. This suggests that these processes are part of the lifestyle of the surface saltmarsh sediment microbial community. Indeed, the most abundant genus in t0 samples was *Desulforsarcina*, representatives of which oxidize both MeSH and DMS (Lyimo et al., 2009).

Dimethylsulfide and MeSH consumption and production rates were shown to change during the incubation experiments, see above, and we now propose explanations for these changes. The percentage of MddA-containing bacteria within MeSH plus C incubations decreased over time, possibly explaining why less MeSH was converted to DMS in these samples compared to t0.

Despite the net reduction in the relative abundance of known DMS consumption genes in the MeSH plus C enrichments, DMS degradation rates increased 5-fold during the incubations. These discrepancies could be due to an increase in the expression levels or activity of the DMS-cycling enzymes described here. Further work involving metatranscriptomics and/or metaproteomics is required to confirm this hypothesis. However, it is also possible that there are bacteria with novel DMS catabolic enzymes in the saltmarsh sediment yet to be discovered. This is supported by the isolation of *Rhodobacterales* bacterium cm12 from this saltmarsh sediment, which degraded DMS but lacked *ddhA, dmoA* and *tmm* genes in its sequenced genome.

There are also likely novel MeSH catabolic genes to be identified since the relative abundance of MTO is vastly reduced in the MeSH plus C incubations compared to t0, despite a 2-fold increase in MeSH degradation rates. Indeed, cultivation-dependent methods yielded multiple *Alpha-* and *Gammaproteobacteria* strains that were not previously suspected to degrade MeSH, but from these only *Spongiibacter* sp. m19 yielded an *mtoX* PCR product with 89% identity at the derived amino acid level to the ratified MTO from *Methylophaga thiooxydans* DMS010 (Eyice et al., 2018). This could be due to either the *mtoX* primers used in this study (Eyice et al., 2018) not covering the diversity of *mtoX* sequences, or that these isolates possess novel pathways to catabolise MeSH. In support of this hypothesis, the genomes of *Labrenzia* sp. mcm14, *Oceanicola* sp. mcm12, *Stappia* sp. mcm29 and *Rhodobacterales* bacterium cm12, isolated from these samples, degrade MeSH but lack *mtoX*.

Novel strains of *Hyphomonas, Vibrio, Alteromonas* and *Marinobacter* able to produce DMS from MeSH were also isolated from Stiffkey saltmarsh sediment. MddA homologs have not been detected in members of these genera, suggesting that these isolates might use alternative enzymes to carry out the Mdd pathway. This would indicate that the Mdd pathway is even more widespread among taxonomically diverse bacteria than previously thought.

Some of the isolates from the saltmarsh sediment were also able to make DMSP and, indeed, *dsyB* genes have been found in *Stappia, Labrenzia* and *Oceanicola* strains (Curson et al., 2017; Williams et al., unpublished). However, no *Vibrio* species have been reported to produce DMSP or contain *dsyB* homologs. Therefore, the molecular characterization of this *Vibrio* strain could lead to the identification of novel genes involved in DMSP production.

Finally, incubations with MeSH significantly increased the relative abundance of *Ponticaulis* and *Methylophaga*, which became the dominant genus in the place of *Desulfosarcina* in the MeSH-only enrichments. Several *Methylophaga* strains from marine environments oxidize both DMS and MeSH (De Zwart et al., 1996; Kim et al., 2007; Schäfer, 2007; Boden et al., 2010). Moreover, Eyice et al. (2018) have shown that *Methylophaga thiooxydans* contains a functional MTO enzyme. The enrichment of *Methylophaga* species harboring the *mtoX* gene explains the significant increase in the relative abundance of *mt*o*X* sequences in the MeSH-only enrichments compared to t0 samples. This suggests that *Methylophaga* is a key MeSH- cycling bacterium in Stiffkey saltmarsh sediment, as it was shown to be for DMS in coastal environments (Neufeld et al., 2008). However, no member of the *Ponticaulis* genus, to our knowledge, have been implicated in MeSH and/or DMS cycling, and strains of *Ponticaulis* for which a genome sequence is available do not have significant homology to known MeSH and/or DMS-cycling genes. It would be interesting to perform stable-isotope probing (Dumont and Murrell, 2005) in the future to ascertain which are the key players in MeSH and DMS metabolism in these samples.

## Conclusion

Here, we show that the Mdd pathway is functional in Stiffkey saltmarsh sediment when MeSH is available, although its activity is masked by Mdd-independent-MeSH and DMS consumption processes. We also demonstrate that MeSH degradation, likely mediated by MTO, is also important in these marine sediment environments. Finally, bacterial strains isolated in this study can be used as model organisms to identify novel genes involved in MeSH and DMS cycling. The identification of novel pathways involved in the metabolism of these volatile compounds and the evaluation of their functionality in the environment are essential steps to better understand the microbial contribution to the global sulfur cycle.

## Data Availability

16S rRNA gene amplicon sequencing and metagenomic data generated in this study are publicly available at NCBI single read archive (BioProject PRJNA450518). 16S rRNA gene sequences of the bacterial strains isolated in this study are deposited in Genbank under accession numbers MH260513 to MH260558. The *mtoX* from strain *Spongiibacter* sp. m19 is available at Genbank (MH271290). *ddhA* genes from *Stappia* sp. mcm29, *Oceanicola* sp. mcm12 and *Labrenzia* sp. mcm14 are available at Genbank under accession numbers MK409622–MK409624.

## Author Contributions

OC and JT designed the experiments, analyzed data and wrote the manuscript with contributions of all authors. OC performed microcosm experiments, isolation and characterization of bacterial strains. JP conducted bioinformatics analysis of 16S rRNA gene amplicon data and metagenomes and prepared figures. KR and MF carried out *mtoX* PCR and qPCR experiments. WR performed statistical analysis on 16S rRNA gene amplicon sequencing data. JM designed experiments and analyzed data.

## Conflict of Interest Statement

The authors declare that the research was conducted in the absence of any commercial or financial relationships that could be construed as a potential conflict of interest.

## Footnotes

1 www.ncbi.nlm.nih.gov

2 http://rdp/cme.msu.edu
