# Supplementary material for: Methanethiol and Dimethylsulfide Cycling in Stiffkey Saltmarsh
Source: Front Microbiol. 2019 May 10;10:1040. doi: 10.3389/fmicb.2019.01040 (PMC6524544; doi:10.3389/fmicb.2019.01040)
Supplement: Supplementary file 1 [file Table_1.docx]

**Supplementary Material**

**Methanethiol and dimethylsulfide cycling in Stiffkey saltmarsh**

Ornella Carrión^1*^, Jennifer Pratscher^2^, Kumari Richa^3^, Wayne G. Rostant^4^, Muhammad Farhan Ul Haque^1^, J. Colin Murrell^1^, Jonathan D. Todd^4*^

Author’s affiliations

^1^ *School of Environmental Sciences, University of East Anglia, Norwich, UK*

*^2^* *The Lyell Centre, Heriot-Watt University, Edinburgh, UK*

*^3^ School of the Environment, Florida Agricultural and Mechanical University, Tallahassee, Florida, USA*

^4^ *School of Biological Sciences, University of East Anglia, Norwich, UK*

^*^Corresponding authors:

Ornella Carrión, ^1^School of Environmental Sciences, University of East Anglia, Earlham Road, Norwich NR4 7TJ, UK.

E-mail: [o.carrion-fonseca@uea.ac.uk](mailto:o.carrion-fonseca@uea.ac.uk)

Jonathan D. Todd, ^4^School of Biological Sciences, University of East Anglia, Earlham Road, Norwich NR4 7TJ, UK.

E-mail: jonathan.todd@uea.ac.uk

**Supplementary Table S1. Selected ratified proteins used to confirm sequences obtained from the metagenomics analysis as functional genes of interest.**

| Refseq | Accession number | Microorganism | Reference |
| --- | --- | --- | --- |
| MddA | AJE75769.1  WP_008148420.1  NP_772381.1  NP_767858.1  YP_001803274.1  NP_217755.1 | *Pseudomonas deceptionensis*  *Pseudomonas* sp. GM41(2012)  *Bradyrhizobium diazzoefficiens* USDA 110  *Bradyrhizobium diazzoefficiens* USDA 110  *Cyanothece* sp. ATCC 51142  *Mycobacterium tuberculosis* H37Rv | Carrión *et al.,* 2015 |
| MTO | ATJ26742.1 | *Hyphomicrobium* sp. VS | Eyice *et al.*, 2018 |
|  | WP_011242048.1 | *Ruegeria pomeroyi* DSS-3 |  |
|  | WP_008290534.1 | *Methylophaga thiooxydans* |  |
| DmoA | E9JFX9.1 | *Hyphomicrobium sulfonivorans* | Boden *et al.*, 2011 |
| DddhA | Q8GPG4.1 | *Rhodovulum sulfidophilum* | McDevitt *et al.,* 2002 |
|  | Q8GPG3.1 |  |  |
| Tmm | ACK52489.1  AAV94838.1  EAQ26624.1 | *Methylocella silvestris* BL2  *Ruegeria pomeroyi* DSS-3  *Roseovarius* sp. 217 | Chen *et al.,* 2011  Lidbury *et al.,* 2016  Lidbury *et al.,* 2016 |


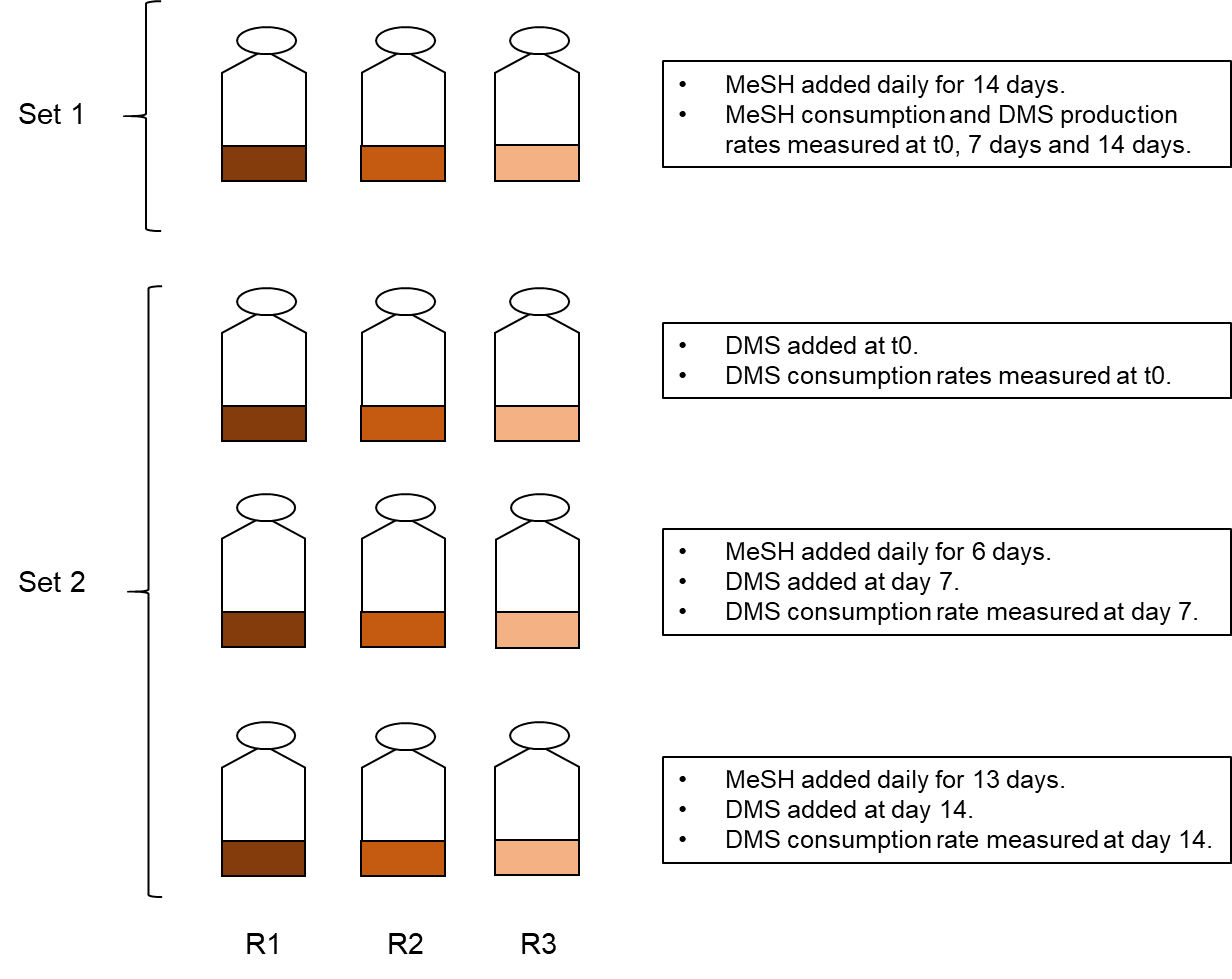


**Supplementary Figure S1. Microcosm experiment set up to measure MeSH consumption and DMS production and consumption rates in surface saltmarsh sediment.** R1: Biological replicate 1; R2: Biological replicate 2; R3: Biological replicate 3.


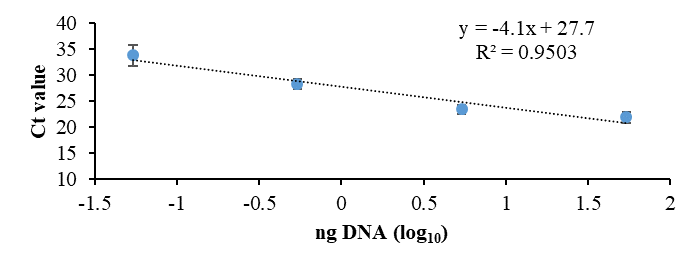


**Supplementary Figure S2. Linear *mtoX* qPCR amplification using 10-fold serial dilutions of DNA extracted from t0 saltmarsh sediment samples.** Each point represents the average of three biological replicates with their respective standard deviations.

**
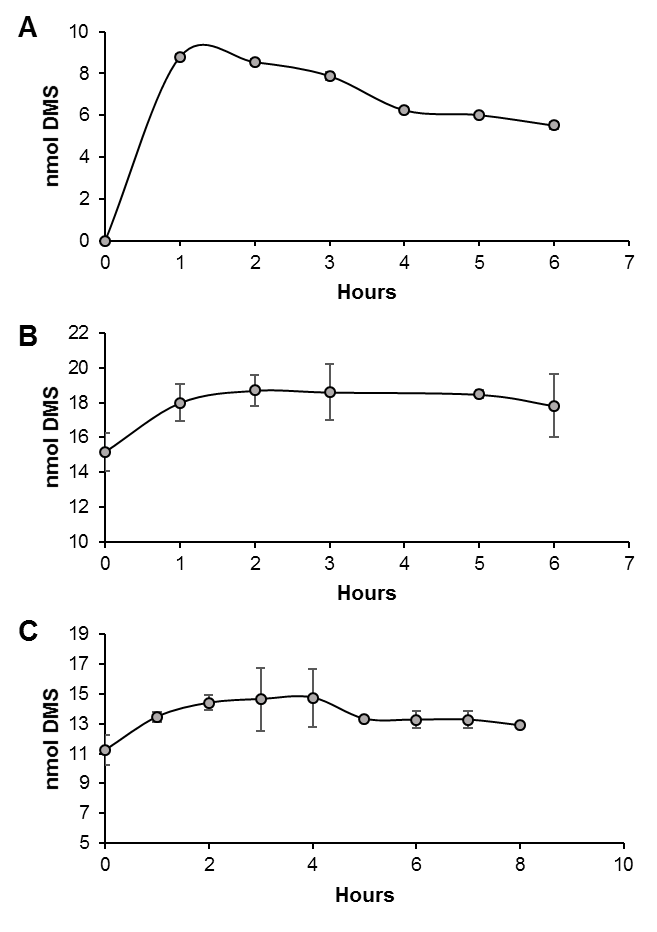
**

**Supplementary Figure S3. DMS production from MeSH by saltmarsh sediment samples. (A):** t0 samples; **(B):** 7-day enrichments with MeSH plus mixed carbon source for 7 days; **(C):** samples enriched with MeSH plus mixed carbon source for 14 days. Each point represents the average of three biological replicates with error bars (smaller than the marker if not visible) showing their respective standard deviations.


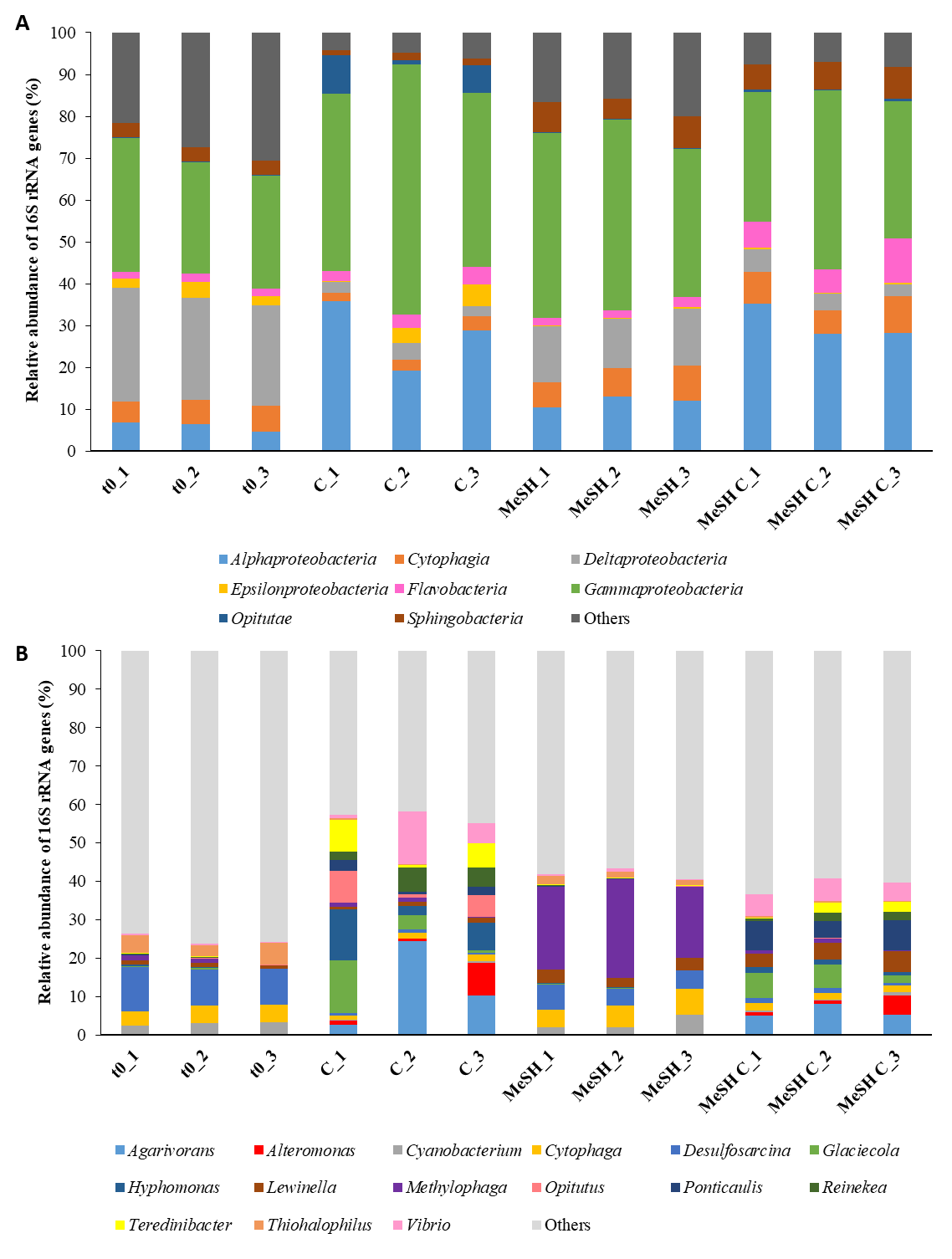


**Supplementary Figure S4. Taxonomic profiling of the 16S rRNA gene amplicon sequencing data from saltmarsh sediment enrichments. (A)** Class level; **(B)** Genus level. Only classes or genera that are ≥5% abundant in at least one of the conditions are represented. t0: natural samples; C: enrichments with mixed carbon source; MeSH: samples enriched with MeSH-only; MeSH C: enrichments with MeSH plus C.

**
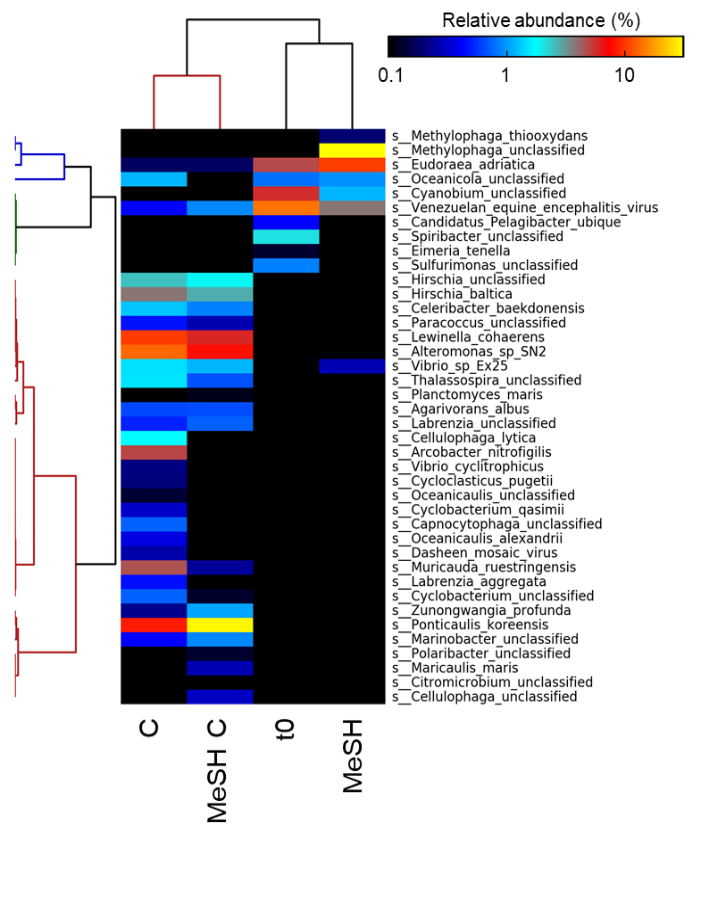
**

**Supplementary Figure S5.** **Phylogenetic analysis of the metagenomes from Stiffkey saltmarsh sediment by MetaPhlAn.** Abundance for species in logarithmic scale reporting the 50 most abundant clades according to the 90^th^ percentile of the abundance of each clade with a custom colour map. Clustering is performed with average linkage, using Bray-Curtis distance for clades and correlation for samples. t0: natural samples; C: enrichments with mixed carbon source; MeSH: samples enriched with MeSH-only; MeSH C: enrichments with MeSH plus C.


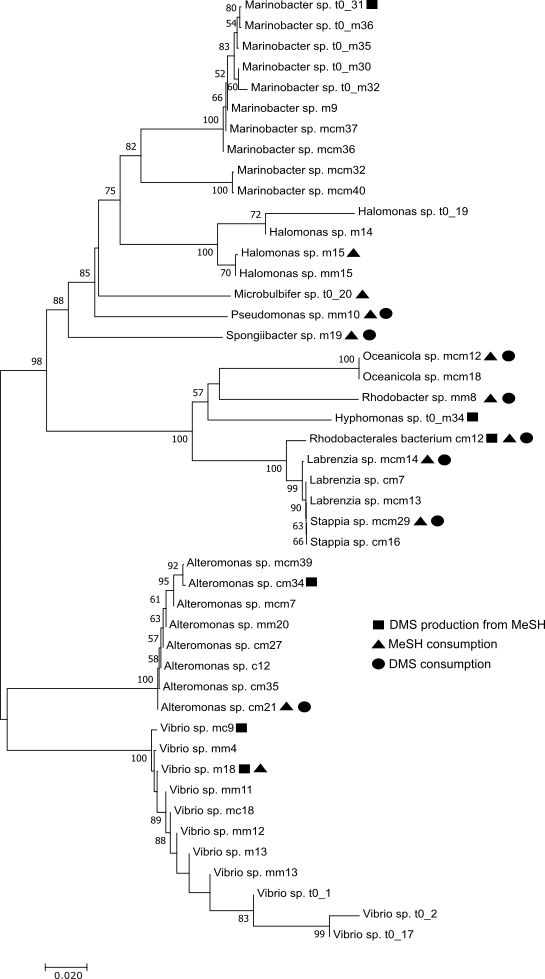


**Supplementary Figure S6. Neighbour-joining phylogenetic tree based on the 16S rRNA gene from bacterial strains isolated from saltmarsh sediment samples.** Bar, 0.02 substitutions per nucleotide position. Bootstrap values ≥50% (based on 1 000 replicates) are shown at branch points.

**References**

Chen Y, Patel NA, Crombie A, Scrivens JH, Murrell JC. (2011). Bacterial flavin-containing monooxygenase is trimethylamine monooxygenase. *PNAS.* 108, 17791-17796.
